# Improving the ability of ED physicians to identify subclinical/electrographic seizures on EEG after a brief training module

**DOI:** 10.1186/s12245-019-0228-9

**Published:** 2019-03-27

**Authors:** Geetha Chari, Kabir Yadav, Daniel Nishijima, Ahmet Omurtag, Shahriar Zehtabchi

**Affiliations:** 1 Department of Neurology, SUNY Downstate Medical Center, Brooklyn, NY USA; 2 Department of Emergency Medicine, Harbor-UCLA Medical Center, Torrance, CA USA; 3 Department of Emergency Medicine, University of California at Davis Health System, Sacramento, CA USA; 4 Nottingham Trent University, Nottingham, UK; 5 Department of Emergency Medicine, SUNY Downstate Medical Center, Brooklyn, NY USA

**Keywords:** Non-convulsive seizures, Emergency department, EEG, Training

## Abstract

**Background:**

Approximately 5% of emergency department (ED) patients with altered mental status (AMS) have non-convulsive seizures (NCS). Patients with NCS should be diagnosed with EEG as soon as possible to initiate antiepileptic treatment. Since ED physicians encounter such patients first in the ED, they should be familiar with general EEG principles as well as the EEG patterns of NCS/NCSE. We evaluated the utility of a brief training module in enhancing the ED physicians’ ability to identify seizures on EEG.

**Methods:**

This was a randomized controlled trial conducted in three academic institutions. A slide presentation was developed describing the basic principles of EEG including EEG recording techniques, followed by characteristics of normal and abnormal patterns, the goal of which was to familiarize the participants with EEG seizure patterns. We enrolled board-certified emergency medicine physicians into the trial. Subjects were randomized to control or intervention groups. Participants allocated to the intervention group received a self-learning training module and were asked to take a quiz of EEG snapshots after reviewing the presentation, while the control group took the quiz without the training.

**Results:**

A total of 30 emergency physicians were enrolled (10 per site, with 15 controls and 15 interventions). Participants were 52% male with median years of practice of 9.5 years (3, 14). The percentage of correct answers in the intervention group (65%, 63% and 75%) was significantly different (*p* = 0.002) from that of control group (50%, 45% and 60%).

**Conclusions:**

A brief self-learning training module improved the ability of emergency physicians in identifying EEG seizure patterns.

## Background

Altered mental status is a common presentation among patients brought in to the emergency department (ED) [1]. Non-convulsive seizures (NCS) and non-convulsive status epilepticus (NCSE) have been detected in approximately 5% of ED patients [2]. Given the prevalence of altered mental status in the ED (2–10%) [1], we estimate that approximately 120,000 to 600,000 ED patients suffer from non-convulsive seizures in the USA annually. NCS and NCSE are serious treatable neurological emergencies, the consequences of which can be severe, in view of the time-dependent survival of seizing neurons. NCS and NCSE are often diagnosed after a substantial delay, often up to 24 h or more after presentation to the ED [3]. This results in delayed initiation of appropriate treatment and worse neurological outcomes. Therefore, it is imperative to diagnose NCS/NCSE early and accurately with electroencephalogram (EEG) and start treatment as soon as possible.

Early ED-based diagnosis and treatment of NCS/NCSE require that an EEG be recorded and interpreted in a timely fashion, as soon as the high risk for NCS/NCSE is determined clinically at the bedside. Since ED physicians are the first to encounter such patients, they should be familiar with the general EEG principles as well as EEG patterns of seizures. Obtaining an emergent EEG in the ED is challenging [4]. Emergent EEGs are still not available in many EDs, especially at nights and weekends. To date, no published study has determined the capacity and availability of stat EEGs in ED across the country. A recent study determined that the use of *micro*EEG™—a miniaturized digital wireless device can be used to acquire an EEG recording rapidly in a busy crowded environment [5]. Once the EEG is being acquired at the bedside, however, the non-expert physician (ED physician) needs to recognize electrographic seizures that require rapid management, especially when access to a trained epileptologist is not possible or delayed.

The objective of this study was to test the utility of a brief training module (a self-learning PowerPoint presentation) to improve the ability of the ED physician to identify electrographic seizures on EEG. This study is a pilot study with a small number of subjects, which will help determine if the EEG training can be expanded and implemented readily.

## Materials and methods

### Study design and setting

This pilot randomized controlled trial was conducted at the departments of emergency medicine of three academic medical centers. All three institutions are academic urban teaching hospitals with emergency medicine residencies. Institutional review boards approved the study in each institution. Informed consent was obtained from all participants prior to enrollment.

### Selection of participants

The trial enrolled board-certified emergency medicine faculty. Physicians with previous EEG training were excluded. Subjects were recruited via email through faculty directories in each institution. The first 10 volunteers in each institution (10 subjects per site, 30 subjects in total) were randomized to control or intervention groups using a random number generating software. Participants were randomized to the intervention group or the control group. Physicians allocated to the intervention group received a self-learning PowerPoint presentation (training module) and were asked to take a quiz after reviewing the PowerPoint presentation. The control group was asked to take the quiz without reviewing the training slides. Two months after the initial date of their initial quiz, the quiz was re-administered without any training slides for either group to test their retention.

#### EEG training module

A slide presentation describing the basic principles of EEG including EEG recording techniques, montages, and views followed by characteristics of normal and abnormal patterns was developed with assistance of epileptologists and experts in educational research. The goal of the presentation was to familiarize the participants with EEG presentations of seizure.

#### Test material

**Fig. 1:**
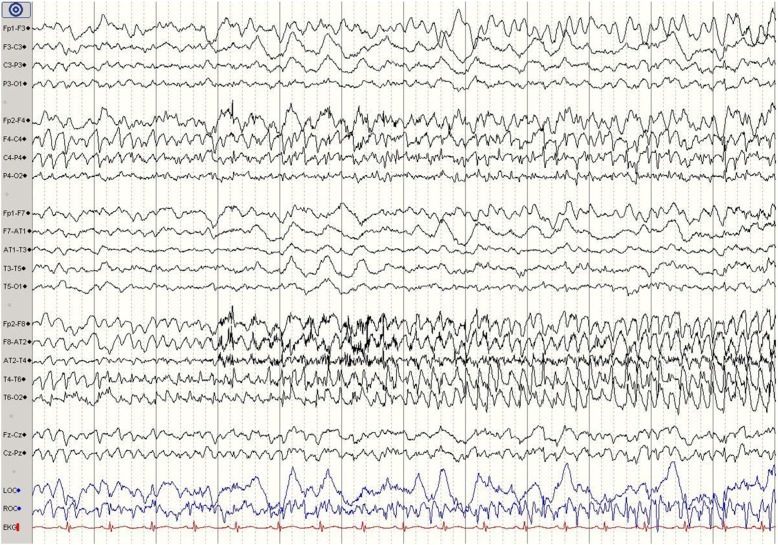
EEG snapshot showing a right temporal focal electrographic seizure

**Fig. 2:**
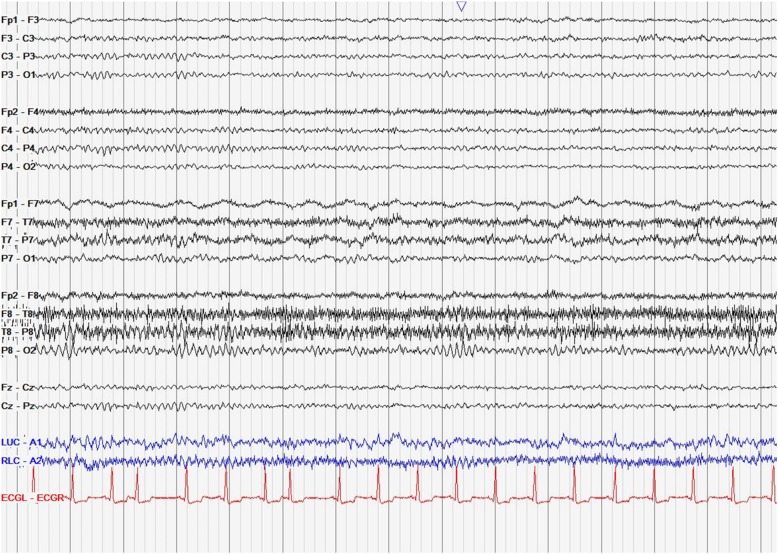
EEG snapshot showing focal slowing over the left temporal region

Participants in both groups were tested in their ability to identify abnormal from normal EEG as well as presence or absence of seizure by reviewing 20 test EEGs (one-page snapshots). These de-identified EEGs were previously recorded from actual patients. Each test EEG was accompanied by two questions: normal or abnormal, and seizure vs no seizure. The test scores range from 0 (all wrong answers) and 40 (all correct answers). The EEG quiz consisted of normal samples (2 slides, wake and sleep), and abnormal samples (18 slides—focal or generalized seizures (9), slowing (3), burst suppression (1), spikes (4), triphasic waves (1)). See examples in Figs. 1 and 2.

Study data were collected and managed using REDCap (Research Electronic Data Capture) electronic data capture tools hosted at Harbor-UCLA Medical Center. REDCap is a secure, web-based application designed to support data capture for research studies, providing (1) an intuitive interface for validated data entry, (2) audit trails for tracking data manipulation and export procedures, (3) automated export procedures for seamless data downloads to common statistical packages, and (4) procedures for importing data from external sources [6].

### Outcome measures

The primary outcome was the percentage of correct answers to the quiz (corresponding to correct interpretation of each EEG snapshot) initially and after 2 months (test of retention).

Method of determination of outcomes: Overall scores and percentages of correct answers were calculated by administering the quiz to all participants. The total number of correct answers for each participant was counted and divided by 40 (maximum score) to calculate the correct score percentage for each subject.

### Statistical analysis

Data are reported as medians and quartiles for continuous variables and percentages with quartiles for proportions. The outcome (percentages of correct answers) was calculated and compared between the two groups using Mann-Whitney *U* test.

We planned a sub-group analysis to compare the responses to seizure versus no seizure questions only between the groups, to specifically examine the performance of physicians to identify seizures on EEG.

## Results

A total of 30 emergency physicians were enrolled (10 per site, 30 in total, 15 controls and 15 interventions). Participants were 63% male with median years of practice of 9 years (quartiles 3, 14). Groups were similar in regards to years of practice and gender (Table 1).

**Table 1:** Comparison of the baseline characteristics of the study groups

Variable	Control group	Intervention group
Gender (female)	8/15	10/15
	53% (95% CI, 30–75%)	67% (95% CI, 42–85%)
Years of experience*	7.5 (3, 14.5)	9 (3, 13.5)

*Median and quartiles

*CI* confidence interval

The percentage of correct answers in the intervention group (65%, quartiles 63% and 75%) was significantly different (*p* = 0.001) from that of control group (50%, quartiles 46% and 59%) for the initial quiz. Similarly, at 2-month follow-up retention quiz, the intervention group performed better than the control group (68% [quartiles 60% and 73%] versus 58% [quartiles 55% and 61%]) but the difference was not statistically significant (*p* = 0.05) (Table 2).

**Table 2:** Comparison of performance of percentages of the correct responses within each group

	Initial quiz*	Follow-up quiz*	*p* value
Control group	50% (46%, 59%)	58% (55%, 61%)	0.325
Intervention group	65% (63%, 75%)	68% (60%, 73%)	0.683

*Median and quartiles

**Fig. 3:**
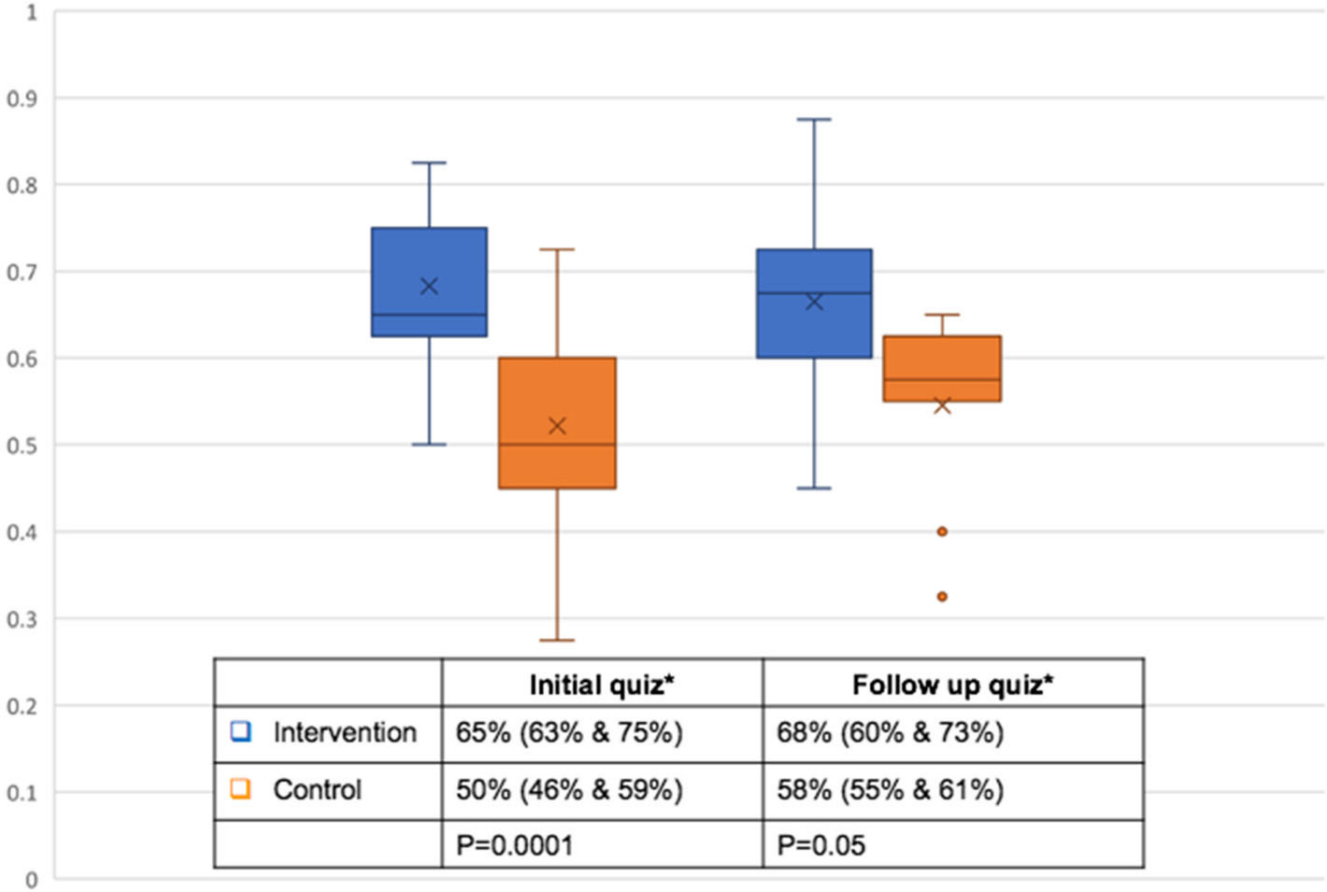
Comparison of percentages of correct answers between control and intervention groups using Box-Whisker plot

Within each group, we did not observe any significant difference between the initial test scores versus the follow-up scores although both groups scored better in the follow-up quiz. The control group scored 50% (quartiles 46% and 59%) correct answers for the initial quiz and 58% (quartiles 55% and 61%) at follow-up quiz (*p* = 0.325). The intervention group’s percentage of correct answers at initial quiz (65% [quartiles 63% and 75%]) and follow-up quiz (68% [quartiles 60% and 73%]) also were not statistically different (*p* = 0.683) (Fig. 3—Box-Whisker plot). Generalized seizures were identified by 80–90% of the subjects. Identification of focal seizures was more variable (between 30 and 80%). Slowing and spikes were over-interpreted as seizures by 60–70% of the subjects.

In the subgroup analysis evaluating the question of seizure versus no seizure, the results were similar to the overall analysis. There was a significant difference between the percentages of correct answers identifying seizures between the intervention group (63%, 95% CI 54–70%) and the control group (45%, 95% CI 40–53%) in the initial quiz. However, this difference was not significant at the time of the follow-up quiz between the intervention group (55%, 95% CI 50–63%) and the control group (50%, 95% CI 45–53%).

## Discussion

The domain of performing and providing EEG interpretation is traditionally with the neurophysiologists/epileptologists. However, with the advent of increasing use of emergent and continuous EEG in various clinical settings (e.g. ICUs, ORs), non-expert clinicians and specialists have to become aware of electrographic seizure patterns which require emergent bedside management, especially when access to an EEG expert is not possible or delayed. In the ED setting, this becomes even more important. The physicians in the ED manage a variety of patients and use several diagnostic modalities to manage patients rapidly. For example, ED physicians evaluate patients with suspected myocardial infarction and are able to recognize critical changes on the electrocardiogram (EKG) at the bedside, even though the EKG is eventually interpreted by the cardiologist. ED physicians also use rapid bedside ultrasound to diagnose and initiate treatment for several conditions [7, 8], while the final confirmatory study and report is provided by the radiologist later. Currently, physicians administer sedatives and anticonvulsants to patients with suspected NCS based on clinical suspicion, without an EEG in most EDs. Training ED physicians to recognize EEG seizures will help them identify and treat NCS appropriately. This will also reduce the risk of administration of anticonvulsants in patients who are not suffering from NCS.

Our study evaluated the efficacy of a PowerPoint EEG training module created by a collaboration of an epileptologist, emergency medicine physicians, and experts in education research to improve recognition of electrographic seizures by ED physicians at the bedside. The purpose of this brief training module was to provide very basic practical clinically relevant knowledge to the physicians, focusing on identifying normal versus abnormal EEG, and the presence or absence of seizures. It was important to include normal patterns besides seizures in the module, as some of these could be misinterpreted as abnormal patterns by an untrained individual. Based on the results, the ED physicians clearly benefited from the training module, as they performed significantly better than the group who were not provided the module. Follow-up assessment in 2 months showed that this group of ED physicians retained that knowledge over time.

Very few studies are present on the review of literature that evaluate educational methods for EEG instruction to non-neurology physicians/residents/fellows. In 2008, Fahy et al. [9] published the results of an EEG learning module in anesthesiology residents, where 40 evaluations were performed on 33 residents. They found that collaborating with the department of neurology to set up an educational module significantly improved the EEG assessment scores among the anesthesiology residents. The same authors [10] published another study in 2014 looking at the long-term retention of a multidisciplinary EEG instructional model for anesthesiology residents and determined that long-term retention was significantly improved after 20 compared to 10 EEG interpretations. In 2010, Chau et al. [11] analyzed the effectiveness of a 45-min EEG educational module in improving assessment scores in nine neurosurgery residents. In this study, the assessment tool scores increased from a mean of 12.00 ± 1.9 before the educational module to 19.7 ± 2.0 (*p* < 0.001).

EEG simulation models have also been developed [12, 13] and appear to provide promising results creating a simulated clinical setting for EEG training. More recently, quantitative EEG and trends have been used to train non-experts in electrographic seizure identification at the bedside [14, 15]. However, no guidelines exist regarding the use of quantitative EEGs and trends.

There are several limitations in this study. The number of study participants is small. No sample size analysis was performed as this was a pilot trial. Follow-up was only at 2 months, and not at a longer interval (e.g. 12 months) to evaluate retention of study material. The participants only interpreted a one-page snapshot of the EEG, which is not representative of the bedside EEG that is recorded for an average of 30 min, if not continuous, and can provide much better visualization of patterns and rhythms. Our study was a pilot study that provides preliminary data. The study module needs further refining and testing, before it can be applied to clinical practice. Determining patient impacts of risks and benefits of treatment of ED patients with NCS is not within the scope of this pilot study. However, our pilot study may justify conducting a larger study to evaluate the safety and efficacy of such a training module in real-time management of patients suspected of NCS.

## Conclusion

This pilot study demonstrates that providing a brief EEG training module can help emergency department (non-neurology) physicians improve the identification of seizures on bedside EEG.

## Abbreviations

AMS: Altered mental status
ED: Emergency department
EEG: Electroencephalogram
NCS: Non-convulsive seizure
NCSE: Non-convulsive status epilepticus
REDCap: Research Electronic Data Capture
